# Establishment and application of a loop-mediated isothermal amplification (LAMP) system for detection of *cry1Ac* transgenic sugarcane

**DOI:** 10.1038/srep04912

**Published:** 2014-05-09

**Authors:** Dinggang Zhou, Jinlong Guo, Liping Xu, Shiwu Gao, Qingliang Lin, Qibin Wu, Luguang Wu, Youxiong Que

**Affiliations:** 1 Key Laboratory of Sugarcane Biology and Genetic Breeding, Fujian Agriculture and Forestry University, Ministry of Agriculture, Fuzhou 350002, China; 2 School of Agriculture and Food Sciences, The University of Queensland, Brisbane 4072, Australia

## Abstract

To meet the demand for detection of foreign genes in genetically modified (GM) sugarcane necessary for regulation of gene technology, an efficient method with high specificity and rapidity was developed for the *cry1Ac* gene, based on loop-mediated isothermal amplification (LAMP). A set of four primers was designed using the sequence of *cry1Ac* along with optimized reaction conditions: 5.25 mM of Mg^2+^, 4:1 ratio of inner primer to outer primer, 2.0 U of *Bst* DNA polymerase in a reaction volume of 25.0 μL. Three post-LAMP detection methods (precipitation, calcein (0.60 mM) with Mn^2+^ (0.05 mM) complex and SYBR Green I visualization), were shown to be effective. The sensitivity of the LAMP method was tenfold higher than that of conventional PCR when using templates of the recombinant *cry1Ac* plasmid or genomic DNA from *cry1Ac* transgenic sugarcane plants. More importantly, this system allowed detection of the foreign gene on-site when screening GM sugarcane without complex and expensive instruments, using the naked eye. This method can not only provide technological support for detection of *cry1Ac*, but can also further facilitate the use of this detection technique for other transgenes in GM sugarcane.

Sugarcane (*Saccharum* spp. hybrids), with a total of 19.4 million hectares in world production^1^, is not only the major source of sugar but is also a promising industrial raw material to produce biofuel due to its high biomass^2^. In China, it accounts for 90–92% of the total sugar output during the last fifteen years^3^. Traditional sugarcane breeding requires a large segregated population for selection, normally reaching 200,000 seedlings for one variety and taking more than ten years to identify and release a new variety^4^,^5^, which is a significant investment in any breeding program. However, the major cultivars are still the ROC series (bred by the Chinese Taiwan Sugarcane Research Institute), among which the variety ROC22 totals about 50% of the total area of sugarcane cultivated in mainland China during the last ten years. The current commercial sugarcane cultivars are interspecies hybrids with allopolyploidy and aneuploidy^6^,^7^ with a large, variable genome size of 3.05–8.91 pg/2C^6^; thus, sugarcane improvement by cross breeding may have reached its potential^7^. Genetic engineering offers an efficient alternative in sugarcane breeding, because it shortens the breeding period and reduces the cost^8^. Along with other advantages, such as asexual reproduction and a smaller possibility of gene drift because of rare flowering and seeding, genetic engineering in sugarcane has the lowest security risk (level I) compared with other crops^7^.

Stem borer is the most harmful pest in most countries that cultivate sugarcane, resulting in extraordinary agricultural and industrial losses^9^. In China, for example, stem borer not only reduces the sucrose content and cane yield, but also dramatically decreases the ratooning ability of sugarcane. Due to its feeding behavior (the larvae crawls into the sugarcane stem), controlling stem borer is difficult, expensive, and has negative environmental impacts (the use of toxic pesticides)^10^. To increase sugarcane production, breeding a borer-resistant cultivar has become a priority. Introducing a foreign borer-resistant gene may be the only option since the borer resistance trait appears to be absent in the gene pool of sugarcane cultivars^10^. Genetic transformation using insecticidal *Bt* or *cry* genes is an effective method to develop insect-resistant transgenic plants, such as commercial genetically modified (GM) soybean (*Glycine max*), cotton (*Gossypium hirsutum*), corn (*Zea mays*) and other crops^11^,^12^,^13^,^14^,^15^. Previous research also revealed that the introduction of the *cry1Ac* gene is an economic and effective strategy to improve the borer-resistant characteristics of sugarcane^16^. The ability to perform effective, sensitive, accurate and reliable identification of transgenic sugarcane plants with the *cry1Ac* gene is essential and important for sugarcane molecular breeding and its commercialization.

Numerous biosafety and risk assessments of transgenic sugarcane lines, including GM sugarcane carrying the *cry* gene, have been conducted in several countries^17^. Since the first GM sugarcane was approved in 2002 in China, up to more than twenty GM sugarcane events including *cry* gene serials have been approved and been examined for biosafety and risk assessment^7^. It is necessary to develop a rapid, stable and low-cost technique for early identification and tracking of foreign genes in transgenic sugarcane for supervision and administration purposes. It would be even more advantageous if the method could be conducted in an ordinary laboratory or even in the field.

A number of methods for genetically modified organism (GMO) detection have been developed, mainly based on nucleic acid amplification such as conventional PCR, competitive PCR, real-time PCR and Southern blot, or based on protein detection, such as Western blot and enzyme-linked immunosorbent assay^18^,^19^. The former is used more frequently because of simple, convenient and relatively higher stability of nucleic acids in contrast to protein-based assays^20^. Currently, conventional PCR remains the most popular laboratory technique but is greatly limited by the need for expensive thermal cyclers, gel electrophoresis systems, gel imaging systems and the use of hazardous ethidium bromide (EB) as fluorescent intercalators^19^,^21^.

Several molecular methods, including loop-mediated isothermal amplification (LAMP), invader, ligase chain reaction (LCR), nucleic acid sequence-based amplification (NASBA), strand displacement amplification (SDA), multiple displacement amplification (MDA) and rolling circle amplification (RCA) have already been introduced into routine detection or are currently under investigation for their performance in pathogen diagnosis and GMO detection^22^,^23^,^24^. Among these methods, LAMP, originally developed by Notomi et al.^24^, has shown to be the most efficient and specific method^23^,^24^. It is specially designed with a set of two outer primers (F3 and B3) and two inner primers (FIP and BIP). This technique is able to recognize six distinct sites flanking the target DNA sequences^18^,^21^,^25^. Since LAMP, which is simple, fast and provides visible products, can be conducted in constant temperature conditions without expensive PCR and DNA detection equipment^21^,^24^. It has been widely adopted and further developed for detection of various pathogens including bacteria, viruses and protozoans^26^,^27^,^28^,^29^,^30^,^31^, and detecting transgenes in GMOs^32^,^33^,^34^,^35^.

Recently, a series of detection methods based on LAMP have been developed for specific GM crops. Fukuta et al.^18^ detected the *CaMV35S* promoter in Roundup-Ready soybean using LAMP, finding that GMO content ranged from 0.5% to 5.0%. Lee et al.^32^ used LAMP in assessing transgenic *MS8* and *RF3* oilseed rape (*Brassica napus*) and GM-related sequences *P-35S*, *P-nos* and *T-nos* from *Agrobacterium* spp. and found that the detection limiting rate of this method was 0.01%, as well as observed the ability of LAMP to work in conditions normally inhibitive to PCR. Chen et al.^36^ also detected *CaMV35S* in GM maize and found that the detection limit of the plasmid DNA in LAMP for *pat* gene was about 100 copies/tube which is higher than that (6 copies/tube) previously reported by Lee et al.^32^ Li et al.^35^ developed a LAMP method for GM rice (*Oryza sativa*) for detection of the exogenous *cry1Ab* gene and found that the detection limit of this assay was as low as 300 copies of plasmid with a detection limiting rate of 0.5%.

There has not been any use of the LAMP method for the detection of transgenic sugarcane. Considering the complex genetic background of modern sugarcane, which is multiploid, aneuploid and has a large chromosomal number (~120), combined with the ongoing commercialization of GM sugarcane, it is critical to develop an easy LAMP detection method for GM sugarcane. In the present study, we aimed to develop an effective, sensitive, accurate and reliable detection method for *cry1Ac* transgenic sugarcane based on the LAMP methodology to meet the high demand for transgenic sugarcane breeding, gene composition inspection and supervision. The LAMP technique established here can be easily extended for use in the detection of GM sugarcane with other target genes.

## Results

### Construction and identification of the plasmid 1Ac0229

In this study, we constructed a plasmid 1Ac0229 recombined with the specific sequence of the *cry1Ac* gene*.* Using the primer pair of *cry1Ac*-3F and *cry1Ac*-3R, a 610 bp product shown in Fig. S1 was amplified, indicating the successful incorporation of the *cry1Ac* gene into the plasmid. The primers F3 and B3 worked equally well in PCR amplification and the expected 210 bp product size was observed (Fig. S1). We also found that the primer pair of F3 and B3 is more efficient than the primer pair of *cry1Ac*-3F and *cry1Ac*-3R.

### Optimization of the LAMP reaction

**Figure 1:**
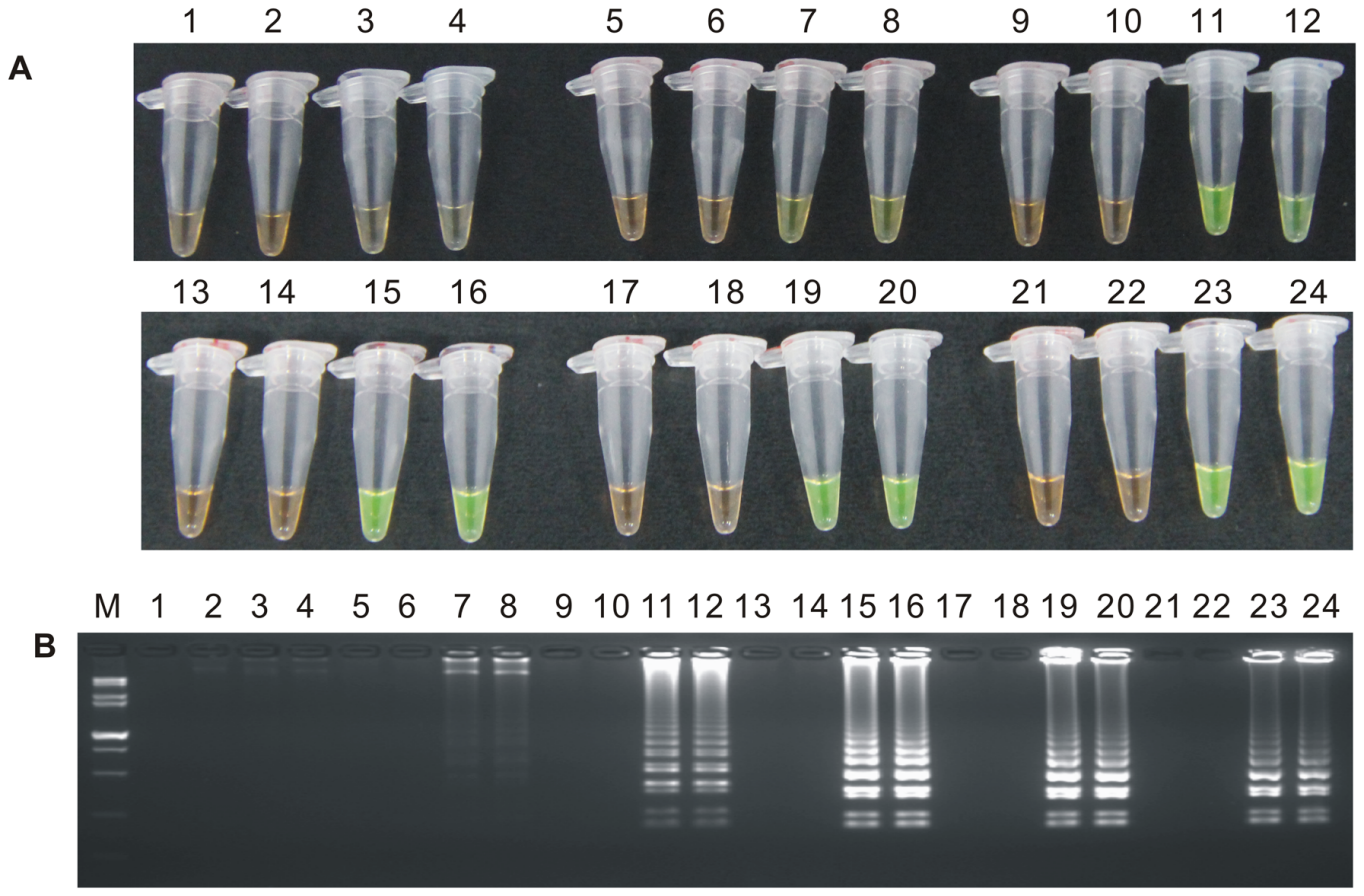
The detection results based on different Mg^2+^ concentrations. (A) LAMP products detected by 1,000× SYBR Green I. (B) Detection of LAMP products by agarose gel electrophoresis stained by EB (ethidium bromide). Tubes and lanes 1, 5, 9, 13, 17 and 21: ddH_2_O. Tubes and lanes 2, 6, 10, 14, 18 and 22: FN95-1702 (negative control). Tubes and lanes 3, 4, 7, 8, 11, 12, 15, 16, 19, 20, 23 and 24: the plasmid 1Ac0229. Tubes and lanes 1–4, 5–8, 9–12, 13–16, 17–20 and 21–24: Concentration of Mg^2+^ is 4.50 mM, 4.75 mM, 5.00 mM, 5.25 mM, 5.50 mM and 5.75 mM, respectively. Lane M: DL 15,000+ 2,000 DNA marker.

**Figure 2:**
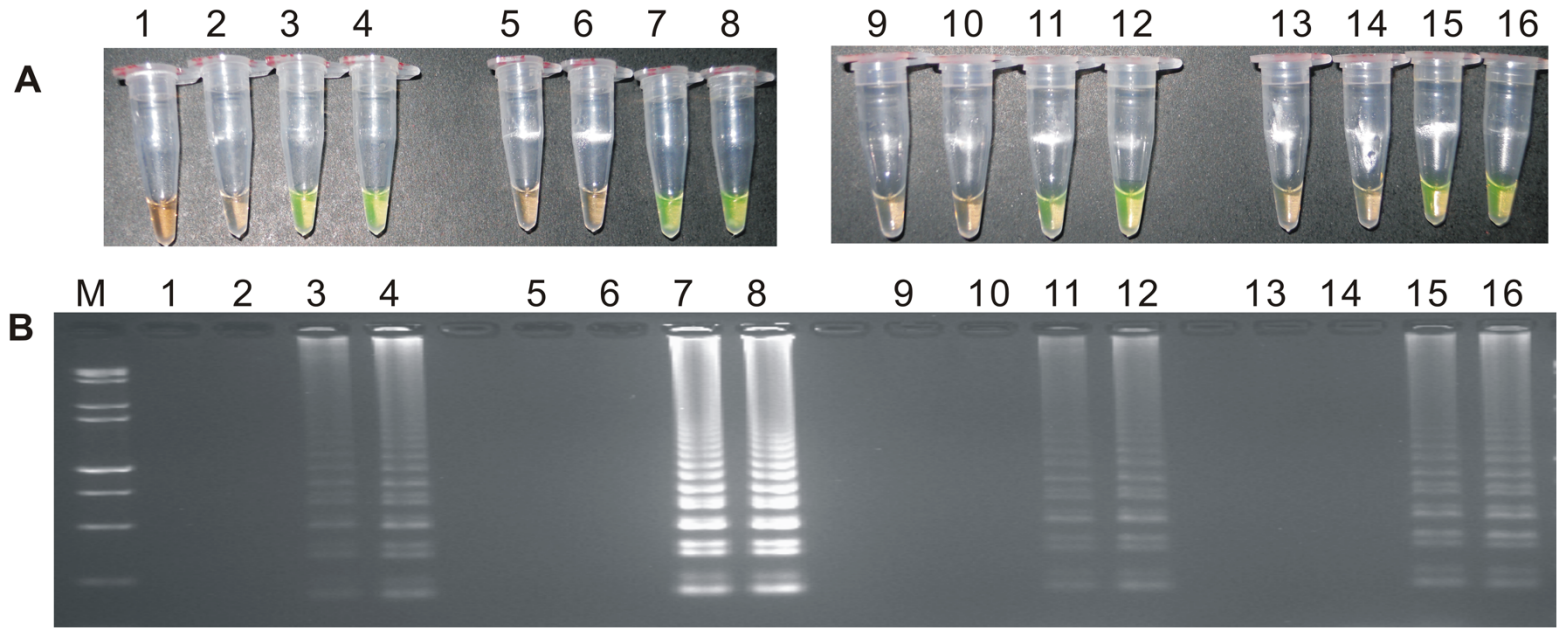
The detection results based on different ratios of inner and outer primers. (A) LAMP products detected by 1,000× SYBR Green I. (B) Detection of LAMP products by agarose gel electrophoresis stained by EB. Tubes and lanes 1, 5, 9 and 13: ddH_2_O. Tubes and lanes 2, 6, 10 and 14: FN95-1702 (negative control). Tubes and lanes 3, 4, 7, 8, 11, 12, 15 and 16: the plasmid 1Ac0229. Tubes and lanes 1–4: ratio of inner and outer primers is 2: 1. Tubes and lanes 5–8: ratio of inner and outer primers is 4: 1. Tubes and lanes 9–12: ratio of inner and outer primers is 6: 1. Tubes and lanes 13–16: ratio of inner and outer primers is 8: 1. Lane M: DL 15,000+2,000 DNA marker.

**Figure 3:**
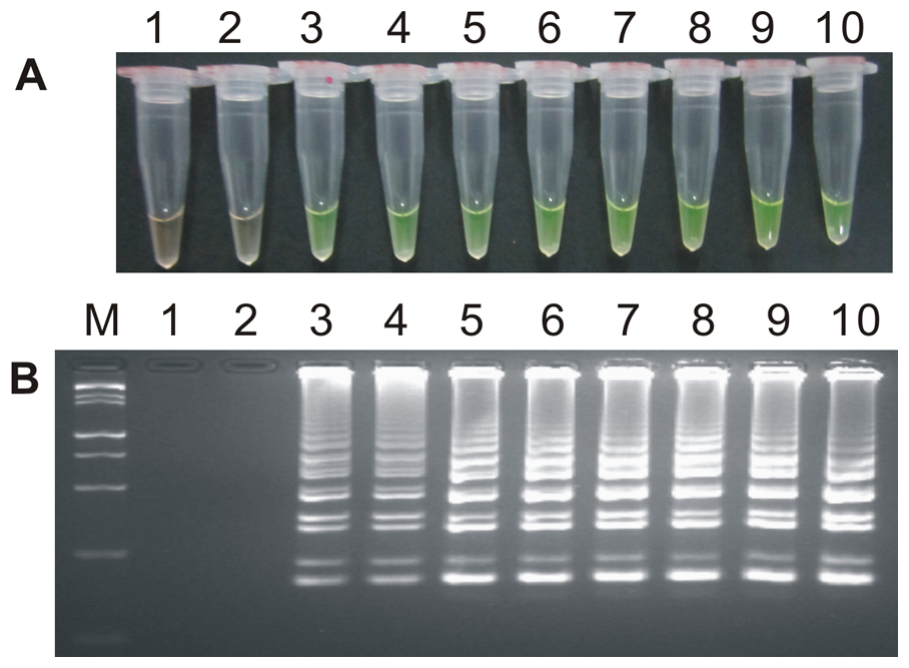
The detection results based on different concentrations of *Bst* DNA polymerase. (A) LAMP products detected by 1,000× SYBR Green I. (B) Detection of LAMP products by agarose gel electrophoresis stained by EB. Tube and lane 1: ddH_2_O. Tube and lane 2: FN95-1702 (negative control, CK). Tubes and lanes 3–4, 5–6, 7–8 and 9–10: *Bst* DNA polymerase concentrations 2.0 U, 4.0 U, 6.0 U and 8.0 U, respectively, two repeats. Lane M: DL 15,000+2,000 DNA marker.

During the optimization of the LAMP system, initial LAMP products were identified by gel extraction and sequencing (data not shown). The effects of Mg^2+^ concentration, *Bst* DNA polymerase amount, concentration ratio between inner and outer primers, and the effect of the addition of 0.80 M betaine on the LAMP reaction were tested (Fig. 1, 2, 3 and S2).

When the concentration of Mg^2+^ was between 4.75 mM and 5.75 mM, the ratio of inner to outer primers was under 2:1, 4:1, 6:1 and 8:1, and the dosage of *Bst* DNA polymerase ranged from 2.0 U to 8.0 U in LAMP, the tubes containing the template plasmid 1Ac0229 with the target sequence of *cry1Ac* gene turned a yellowish green, while the tubes without plasmid 1Ac0229 remained orange (Fig. 1A, 2A and 3A). Agarose gel electrophoresis (AGE) showed visible ladder-like DNA fragments (Fig. 1B, 2B and 3B). However, there were more intense ladder-like bands at the concentration ratio of 4:1 (Fig. 2B) and the amplification improved obviously as the dosage of *Bst* DNA polymerase increased from 2.0 U to 8.0 U, as evidenced by brighter bands on agarose gels (Fig. 3B). It is should be noted that no significant difference was observed in the LAMP reaction with or without the addition of 0.80 M betaine (Fig. S2).

The above results demonstrated that the optimized LAMP reaction consisted of Mg^2+^ at a concentration of 5.25 mM, the ratio of inner to outer primers of 4:1, and the concentration of *Bst* DNA polymerase of 2.0 U if reducing cost is considered.

### Sensitivity of LAMP and conventional PCR

**Figure 4:**
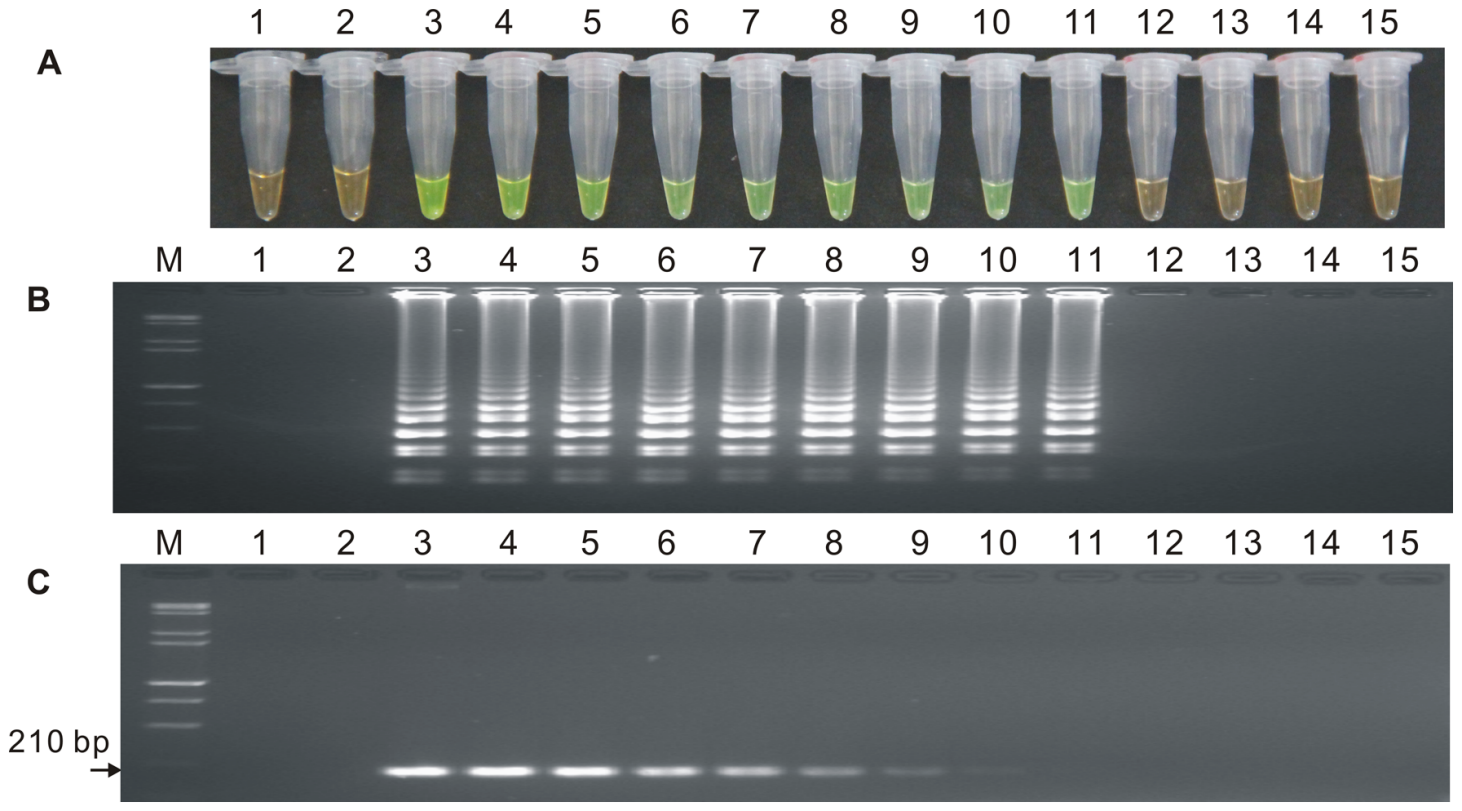
Sensitivity comparison of the LAMP assay and conventional PCR using the plasmid 1Ac0229 as template. (A) LAMP products detected by 1,000× SYBR Green I. (B) Detection of LAMP products by agarose gel electrophoresis stained by EB. (C) PCR products detected by agarose gel electrophoresis stained by EB. Tube and lane 1: ddH_2_O. Tube and lane 2: FN95-1702 (negative control, CK). Tubes and lanes 3–15: plasmid 1Ac0229 copies of 4.31 × 10^9^, 4.31 × 10^8^, 4.31 × 10^7^, 4.31 × 10^6^, 4.31 × 10^5^, 4.31 × 10^4^, 4.31 × 10^3^, 4.31 × 10^2^, 4.31 × 10^1^, 4.31 × 10^0^, 4.31 × 10^−1^, 4.31 × 10^−2^ and 4.31 × 10^−3^, respectively. Lane M: DL 15,000+ 2,000 DNA marker.

**Figure 5:**
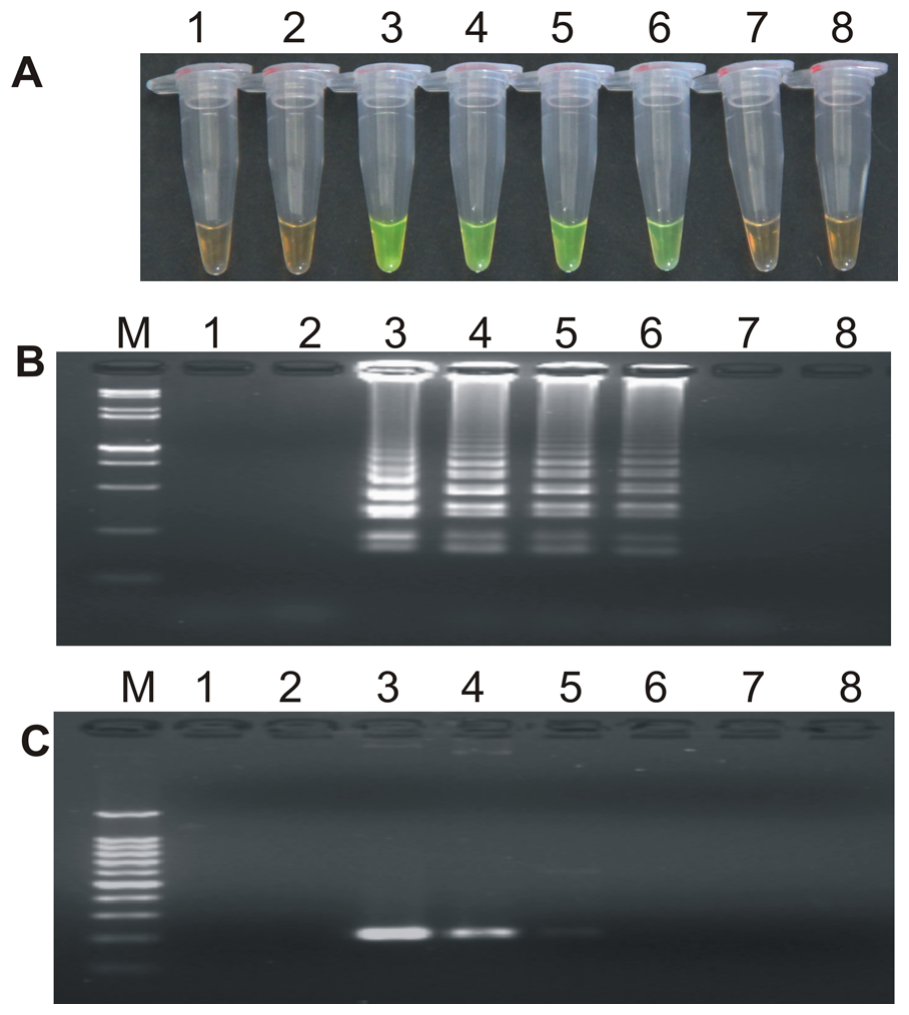
Sensitivity comparison of the LAMP assay and conventional PCR using the gDNA of the *cry1Ac* transgenic sugarcane line 19a-1 as template. (A) LAMP products detected by 1,000× SYBR Green I. (B) Detection of LAMP products by agarose gel electrophoresis stained by EB. (C) PCR products detected by agarose gel electrophoresis stained by EB. Tube and lane 1: ddH_2_O, tube and lane 2: FN95-1702 (negative control, CK), tube and lane 3: the plasmid 1Ac0229, tubes and lanes 4–8: 19a-1 gDNA concentrations 100.0 ng·µL^−1^, 10.0 ng·µL^−1^, 1.0 ng·µL^−1^, 0.1 ng·µL^−1^ and 0.01 ng·µL^−1^, respectively. Lane M: DL 15,000+2,000 DNA marker in B, 100 bp DNA marker in C.

The sensitivities of the LAMP assay and conventional PCR were compared using ten-fold serial dilutions of the plasmid 1Ac0229 (Fig. 4) and genomic DNA from the 19a-1 line as templates (Fig. 5), respectively. The results from AGE (Fig. 4B, 4C, 5B and 5C) revealed that the detection limit of the LAMP method was 43.1 copies of plasmid (Fig. 4B) and 1.0 ng·µL^−1^ sugarcane genomic DNA (Fig. 5B), while that of the conventional PCR method was 431 copies (Fig. 4C) and 10.0 ng·µL^−1^ (Fig. 5C), respectively.

These results suggested that the sensitivity of LAMP was around 10 times higher than that of conventional PCR, independent of whether the test template was plasmid 1Ac0229 or genomic DNA from transgenic sugarcane line 19a-1.

### Comparison of analysis methods for LAMP products

**Figure 6:**
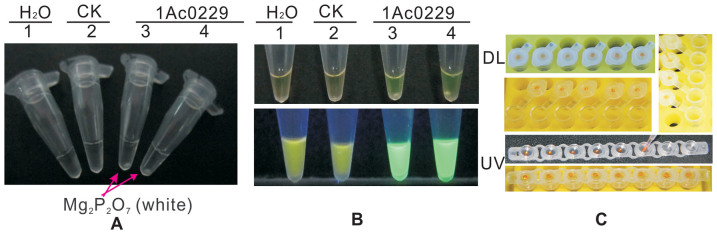
Comparison of detection methods for LAMP products. (A) Visual assessment based on white precipitation (Mg_2_P_2_O_7_). (B) Calcein and Mn^2+^ complex assessment, *top* *panel* under daylight (DL), *bottom panel* under ultraviolet (UV). (C) Modified SYBR Green I method (2 µL 1,000× SYBR Green I be dropped at the center of the tube caps before the reaction). Tube 1: ddH_2_O. Tube 2: FN95-1702 (negative control, CK). Tubes 3 and 4: the plasmid 1Ac0229.

Other frequently used detection methods, including precipitation observation, AGE, SYBR Green I and modified SYBR Green I method, calcein and Mn^2+^ complex, and detection under ultraviolet light at a wavelength of 365 nm, were compared. After a brief centrifugation, the positive reading is visible to the naked eye in the precipitation observation of the white precipitate of magnesium pyrophosphate (Mg_2_P_2_O_7_) (Fig. 6A). AGE could be used as an efficient supplementary means to visualize LAMP products while establishing the LAMP system (Fig. 4B and Fig. 5B). However, the application of AGE is limited by the use of the highly hazardous chemical EB and the requirement of an expensive imaging system. Results also revealed the feasibility of SYBR Green I (Fig. 1A, 2A, 3A and S2) and a modified SYBR Green I method (Fig. 6C), as well as the calcein and Mn^2+^ complex method (Fig. 6B). Since SYBR Green I and calcein and Mn^2+^ complex are based on a color reaction, both are easier ways to detect LAMP products than that of precipitation observation and AGE. The detection under ultraviolet light (wavelength 365 nm) was also shown to be an effective supplementary method as the obvious difference between positive and negative tubes could be observed objectively.

### Detection of putative *cry1Ac* transgenic sugarcane clones by LAMP and conventional PCR

**Figure 7:**
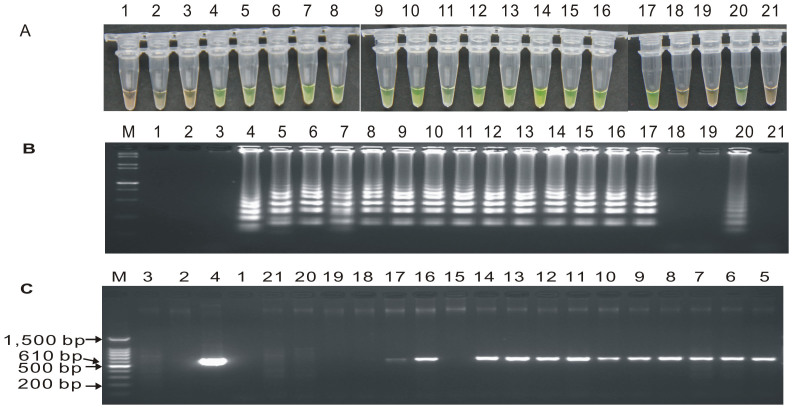
LAMP and conventional PCR detection results of seventeen putative *cry1Ac* transgenic sugarcane lines. (A) LAMP products detected by 1,000× SYBR Green I. (B) Detection of LAMP products by agarose gel electrophoresis stained by EB. (C) PCR products detected by agarose gel electrophoresis stained by EB. Tube and lane 1: ddH_2_O. Tube and lane 2: FN95-1702 (negative control). Tube and lane 3: ROC22 (negative control). Tube and lane 4: the plasmid 1Ac0229 (positive control). Tubes and Lanes 5–21: the seventeen putative *cry1Ac* transgenic sugarcane in order of 16k-1, 16k-3, 16k-5, 16d-1, 16d-2, 16d-4, 16d-6, 19a-1, 19a-3, 19a-5, 19b-4, 20i-2, 20i-4, A-2, A-5, B-2 and B-5. Lane M: 100 bp DNA ladder.

To test the reliability of the LAMP reaction system optimized in this study, the developed protocol was applied to seventeen putative transgenic *cry1Ac* sugarcane lines shown to be resistant to phosphinothricin (PPT). Meanwhile, conventional PCR was also used for all these lines. In the LAMP assay, orange color reactions were observed in three putative transgenic lines, A-2, A-5 and B-5 (in Fig. 7A, tube 18, 19 and 21, respectively), as well as in the blank control and negative controls, while the remaining fourteen tubes and positive control tube displayed a yellowish green color, indicating that the three transgenic lines A-2, A-5 and B-5 may be false-positive. This is probably due to escaping of PPT selection at the stage of in vitro culture screening or due to these lines only being incorporated with the PPT-resistant gene *bar* but not the *cry1Ac* gene. These results demonstrated that the fourteen putative transgenic lines 16k-1, 16k-3, 16k-5, 16d-1, 16d-2, 16d-4, 16d-6, 19a-1, 19a-3, 19a-5, 19b-4, 20i-2, 20i-4 and B-2, were positive for the *cry1Ac* gene (Fig. 7A and 7B, tubes and lanes 5–17 and 20).

In the conventional PCR assay, the specific 610 bp product was amplified in the reactions when the genomic DNA of transgenic lines 16k-1, 16k-3, 16k-5, 16d-1, 16d-2, 16d-4, 16d-6, 19a-1, 19a-3, 19a-5, 20i-2 and 20i-4 were used (Fig. 7C, lanes 5–14,16 and 17), though line 20i-4 (Fig. 7C, lane 17) had relatively lower amounts of target amplified products. No PCR product was observed in the other remaining five putative regeneration lines 19b-4, A-2, A-5, B-2 and B-5 (Fig. 7C, lanes 15, 18–21).

In order to further detect the reliability and specificity of the LAMP assay in transgenic sugarcane, we used quantitative TaqMan real-time PCR to identify the copy number of the *cry1Ac* gene (results shown in Fig. S3 and Table S1) and used quantitative ELISA to measure Cry1Ac protein expression (shown in Table S2). The quantitative TaqMan real-time PCR results showed that all the lines except A-2, A-5 and B-5 contained the *cry1Ac* gene with copy numbers ranging from 1 to 135, which is in agreement with the results by the quantitative ELISA. Therefore, the detection results obtained by LAMP, quantitative TaqMan real-time PCR and quantitative ELISA were consistent. For the seventeen putative transgenic sugarcane lines, there was only a difference in detection in 19b-4 and B-2. Lines 19b-4 and B-2 showed negative results in the conventional PCR assay but positive in LAMP assay, quantitative TaqMan real-time PCR and quantitative ELISA, indicating that the LAMP assay developed in this study had better specificity and higher sensitivity than conventional PCR.

## Discussion

In the present study, we developed a rapid visual LAMP assay for transgenic sugarcane. Compared to the optimized conventional PCR, which requires 3 hours of reaction time, expensive and specialized equipment such as a thermal cycler, a gel system and a gel scanner, the LAMP technique is a relatively simple, feasible, time saving (only 1 hour) and cost-efficient alternative^19^. These features are very important for any successful detection method, especially for those genes absent in the sugarcane gene pool. To our knowledge, this is the first instance of a LAMP assay developed for the detection of *cry1Ac* transgenic sugarcane.

In our study, a LAMP assay was developed and optimized with a set of four specifically designed primers capable of recognizing a total of six distinct regions on the target *cry1Ac* gene. To enhance the reliability of this technique, a complete control system was introduced including a positive control with the plasmid 1Ac0229 containing the *cry1Ac* gene, a negative control with the genomic DNA from non-transgenic parent varieties and a blank control of water. Furthermore, the specificity and sensitivity of the LAMP assay was further supported by conventional PCR. Nagamine et al.^37^ accelerated the LAMP reaction by using loop primers, which suggested that the LAMP reaction times would be shorter than the original method when using loop primers. Though we tried to design loop primers using the PrimerExplorer 4.0 software (http://primerexplorer.jp/e/) based on the selected best group primers from the designed primers of all five groups (data not shown), unfortunately no appropriate loop primers could be found due to lack of suitable loop B (LB). Based on the manual for LAMP primer designing (http://primerexplorer.jp/e/v4_manual/pdf/PrimerExplorerV4_Manual_1.pdf), the loop primers are not an essential requirement for LAMP, so the LAMP reaction performed in the present study did not use loop primers.

Because the size of target DNA affects the reaction time of LAMP^24^, this study utilized a 1 hour reaction time for the LAMP assay in accordance to the size of our target DNA (210 bp)^34^. Previous studies revealed that 0.80 M betaine resulted in elevated sensitivity and increased effectiveness of the LAMP assay^28^,^29^,^30^. However, Chen et al.^38^ found that betaine had no beneficial effect on LAMP amplification. Our study also showed that 0.80 M betaine had no effect on LAMP amplification. Betaine is assumed to be capable of promoting GC-rich DNA amplification and preventing secondary structure formation in GC-rich regions, due to reduction of base stacking^38^. However, our target sequences (GC% = 51.6%) belong to non-GC-rich target sequences, which may be a reason why the addition of 0.8 M betaine had no effect on the LAMP assay in this study, indicating that betaine is not an essential requirement when amplifying non-GC-rich target sequences.

Conventional PCR is normally used in the detection of transgenes due to low-cost and simple procedures compared with real-time qPCR. In this study, using a series of dilutions of either plasmid DNA or genomic DNA from *cry1Ac* transgenic sugarcane, the developed LAMP method had around ten-fold higher sensitivity than that of the conventional PCR. Similar results were observed by Chen et al.^36^ for detection of *CaMV35S* in GM maize, with a limit of the recombinant plasmid pGreen0229-*35S-CBF3-DHA* as low as 6.5 fg in the LAMP assay and 65 ~ 650 fg in the conventional PCR. Using the LAMP assay developed in the present study, we detected two transgenic lines that escaped detection from the conventional PCR assay, out of the 17 putative *cry1Ac* transgenic sugarcane lines derived from 4 different varieties. All of above results confirmed that the LAMP assay developed in this study has a higher sensitivity than that of the conventional PCR.

The high specificity and sensitivity of LAMP can sometimes lead to false-positive amplification due to cross contamination, caused especially by aerosol in the assay process^21^,^24^,^26^,^34^. In addition, dual filter tips, the closure of reaction tube caps and disposal in plastic bags is recommended whenever possible^21^,^34^. Furthermore, ultraviolet sterilization or 75% ethyl alcohol disinfection and a well-ventilated area may be necessary^21^,^34^. Re-designing primers is recommended if critical contamination occurs^34^.

Multiple methods of product detection produced in the LAMP assay demonstrates its flexibility^21^,^24^,^34^,^39^. Initially, methods of observation of a white precipitate and AGE were used. In our study, we found it was difficult to detect the minute amount of precipitation with the naked eye, especially when the LAMP assay was conducted in the field. Since SYBR Green Ι detection was based on the amplified fluorescence emitted from its binding to the minor groove of double-strand DNA, a higher sensitivity can be expected compared to the formation of the white precipitate. Previous studies revealed that it would reduce product amplification if SYBR Green Ι was added before the reaction, while aerosol contamination would often lead to false-positives when the dye was added after the reaction^21^,^34^. We solved this problem by placing a drop of SYBR Green Ι to the center of the tube caps before the reaction and mixing afterwards. Our system is much simpler and more effective compared to the addition of a microcrystalline wax-dye capsule containing SYBR Green I^39^. After our modification in this study, addition of calcein and Mn^2+^ before the reaction also becomes a simple visual detection method, particularly for laboratory detection. Observation under ultraviolet light and AGE are also acceptable but not recommended due to the hazardous substance EB and harmful ultraviolet light, but they could be used as supplementary examination methods during the development of the LAMP assay.

For the first time in sugarcane plants, a LAMP method was established with a closed-tube for visible detection of *cry1Ac* transgenic sugarcane by adding SYBR Green Ι to the cap center of the tube before the reaction. Using a series of dilutions of either plasmid DNA or genomic DNA from transgenic sugarcane carrying the *cry1Ac* gene, this LAMP method has proven to be efficient, specific and reproducible in the detection of the target transgene under a constant temperature with ten-fold higher sensitivity than that of conventional PCR. Therefore, the optimized LAMP method, which allows detection without the need for specialized equipment, is more suitable for field testing and will further facilitate on-site screening. Furthermore, this modified visualized LAMP method can effectively reduce aerosol contamination, which may introduce false-positives. The success in the establishment of an inexpensive, rapid and visible LAMP protocol for *cry1Ac* transgenic detection is significant for extension of this technique for detection of other transgenes in sugarcane engineering.

## Methods

### Plant materials and genomic DNA

Two non-transgenic sugarcane (*Saccharum* spp.) cultivars, FN95-1702 and ROC22, and seventeen putative *cry1Ac* transgenic sugarcane clones were used as testing materials. Among them, six clones of 19a-1, 19a-3, 19a-5, 19b-4, 20i-2 and 20i-4, were derived from the variety FN95-1702, and seven clones of 16k-1, 16k-3, 16k-5, 16d-1, 16d-2, 16d-4 and 16d-6, were derived from the variety ROC22. A-2 and A-5 were derived from GT 94-119 and the remaining two, B-2 and B-5, were derived from GT96-44. All the above plant materials are provided by the Key Lab of Sugarcane Biology and Genetic Breeding, Ministry of Agriculture, China. The genomic DNA was extracted using the modified CTAB protocol^40^. The DNA quality was assessed by AGE and the DNA purity was determined by calculating the A260/A280 ratio using NanoVue Plus^TM^ (GE, New Jersey, USA). The final DNA concentrations were adjusted to 100 ng·µL^−1^.

### Primer design

Four primers for LAMP, including two outer primers (F3 and B3) and two inner primers (FIP and BIP), which recognize a total of six distinct regions of the 1,840 bp *cry1Ac* gene (GenBank: KF630361.1), were designed using the PrimerExplorer 4.0 software (http://primerexplorer.jp/e/). Primer design chart and primer sequences are shown in Fig. S4 and Table S3, respectively. One set of primers (*cry1Ac*-3F: 5′-GCTTGGAGCGTGTCTGGGGT-3′, *cry1Ac*-3R: 5′-TTCTGTGGTGGGATTTCGTC-3′, the amplification product is 610 bp), and the pair of primers F3 and B3 (the amplification product is 210 bp) were used for the specific detection of *cry1Ac* by PCR. All the primers, of which the purity was of HPLC grade, were synthesized by TaKaRa Biotechnology Co., Ltd., Dalian, China.

### Plasmid 1Ac0229

Plasmid 1Ac0229 which contains the *cry1Ac* gene was extracted from the transformed *Escherichia coli* DH5α strain by Plasmid Mini Kit I (Bio-tek Co., Ltd., Beijing, China), and identified by PCR. The concentration was determined by GE NanoVue Plus^TM^ and the original copy number of this plasmid was 4.31 × 10^9^.

### Reaction mixture for LAMP

The initial condition of the LAMP reaction was adopted from Wang et al.^34^ The initial LAMP was carried out in a 25.0 µL mixture containing 3.75 mM MgSO_4_ (50.0 mM, Sigma-Aldrich Inc., St. Louis, USA), 1.4 mM dNTPs (10.0 mM, TaKaRa Biotechnology Co., Ltd., Dalian, China), 2.5 µL 10× ThermoPol Reaction Buffer (New England Biolabs, Massachusetts, USA, including 20.0 mM Tris-HCl (pH 8.8), 10.0 mM KCl, 2.0 mM MgSO_4_, 10.0 mM (NH_4_)_2_SO_4_, 0.1% Triton X-100), 0.8 µM each FIP and BIP, 0.2 µM each F3 and B3, 8.0 U *Bst* DNA polymerase large fragment (New England Biolabs, Massachusetts, USA) and a specified amount of sugarcane genomic DNA or plasmid 1Ac0229. The mixture was incubated at 65°C for 60 min, followed by heating at 80°C for 5 min to inactivate the enzyme and terminate the reaction. Products were then kept at 4°C.

### Optimization of LAMP

Plasmid 1Ac0229 was used to optimize the reaction and as a positive control, and ddH_2_O sterilized by filtration with 0.2 μm filter membrane (Millipore, Carrigtwohill, Co., Ltd. Cork, Ireland) after 121°C for 1 hour was used as a blank control, while DNA from non-transgenic cultivars FN95-1702 or ROC22 was used as the negative control.

Based on the initial conditions of the LAMP reaction adopted from Notomi et al.^24^, six Mg^2+^ concentrations (4.50 mM, 4.75 mM, 5.00 mM, 5.25 mM, 5.50 mM and 5.75 mM), four *Bst* DNA polymerase concentrations (2.0 U, 4.0 U, 6.0 U and 8.0 U), four concentration ratios between inner to outer primers (2:1, 4:1, 6:1 and 8:1) and two betaine (Aladdin Chemistry Co., Ltd., Shanghai, China) concentrations (without betaine, 0.80 M) were tested in 25.0 µL reaction system, while the concentrations of all the other components remained constant respectively. Three replicates were conducted for each experiment.

### PCR Reaction

For optimized PCR, the reactions were performed using final volumes of 25.0 µL, including 2.5 µL 10 × Ex Taq Buffer (Mg^2+^ Plus), 0.005 mM each dNTP, 0.005 µM each primer, 0.625 U Ex-Taq DNA polymerase (TaKaRa Biotechnology Co., Ltd., Dalian, China), and 1.0 µL template DNA. All of the amplifications were performed using a thermal cycler (Mastercycler Gradient 96, Eppendorf, Germany) with the following parameters: one step of 4 min at 94°C, 35 cycles of 30 s at 94°C, 30 s at 57°C, 30 s at 72°C, and one step of 4 min at 72°C. All PCR products detected by electrophoresis on a 1.0% (w/v) agarose gel containing EB (0.5 µg·mL^−1^) in 1 × TAE buffer (pH 8.0) at 100 V for 1 hour and visualized under UV light.

### Sensitivity comparison between LAMP and conventional PCR

The sensitivities were compared between the optimized LAMP and optimized conventional PCR with tenfold serial dilutions of template DNA, either plasmid 1Ac0229 or 19a-1 genomic DNA. 19a-1 was a known *cry1Ac* transgenic sugarcane line at the nucleotide level and protein level, which was confirmed by southern blot and ELISA, respectively. The initial genomic DNA concentration was 100 ng·µL^−1^, and the original copy number of plasmid 1Ac0229 was 4. 31 × 10^9^.

### Comparison of analysis methods for LAMP products

In our study, the amplified products were detected by color change^34^ by staining with 1,000 × SYBR Green I (Bio-tek Co., Ltd., Beijing, China, dropping 2.0 µL of SYBR Green I at the cap center of each reaction tube before incubation). Samples that turned yellowish green were considered positive, while those remained orange were assumed to be negative^19^. In addition, all LAMP products with an aliquot of 2.0 µL were analyzed by AGE (2.0%, w/v)^21^,^24^,^34^. Presence of ladder-like DNA amplification products was considered as positive reaction of LAMP, while the absence of ladder-like DNA amplification products was considered as negative^24^.

In addition, several frequently used detection methods were compared, including visible precipitation (Magnesium Pyrophosphate, Mg_2_P_2_O_7_) (centrifuge at 3,000 r·min^−1^ for 5 min before observation)^21^,^41^, observation by calcein (Aladdin Chemistry Co., Ltd., Shanghai, China) and Mn^2+^ (MnCl_2_, Sigma-Aldrich Inc., St. Louis, USA) complex (calcein 0.6 mM and Mn^2+^ 0.05 mM)^21^,^42^, and observation under ultraviolet light at a wavelength of 365 nm^21^,^42^,^43^.

### Putative transgenic *cry1Ac* sugarcane clones detected by LAMP and conventional PCR

Seventeen putative transgenic *cry1Ac* sugarcane clones were collected randomly from an experimental station at Fujian Agriculture and Forestry University. The youngest fully expanded leaf, namely +1 leaf, with a visible dewlap (the collar between the leaf blade and sheath), was randomly collected from each putative transgenic *cry1Ac* sugarcane line. All of the seventeen putative *crylAc* transgenic sugarcane lines were detected in both the developed LAMP assay and conventional PCR with a DNA concentration of 10.0 ng·µL^−1^. Three biological replicates and two technical replicates were conducted for each sample.

### Copy number of the putative transgenic *cry1Ac* sugarcane clones by quantitative TaqMan real-time PCR

Quantitative TaqMan real-time PCR was applied to identify the copy number of *cry1Ac* gene. Sequences of the primers and probe are: *cry1Ac*-F: ACCGGTTACTCCCATCGA, *cry1Ac*-R: CCAGCACCTGGCACGAA; *cry1Ac*-Probe: 5′ -FAMTCTCCTTGTCCTTGACACAGTTTCTGCTCA TAMRA-3′. Quantitative TaqMan real-time PCR assays were performed on an ABI 7500 System (Applied Biosystems, Foster, USA) using final volumes of 25.0 µL, consisting of 12.5 μL FastStart Universal Probe Master, 0.20 μL probe (10.0 μM), 2.25 μL primer each (10.0 μM), and 1.0 µL template DNA (25.0 ng·µL^−1^) with the following conditions: 50°C 2 min; 95°C 10 min; 40 cycles of 95°C 15 s, 60°C 1 min. Each of the purified 1Ac0229 plasmids was diluted with sterile deionized water to obtain a standard series from 1.0 × 10^8^ to 1.0 × 10^1^ copies per μL with each step differing by tenfold. It is necessary to suspend well by pipetting 30 times when diluting. Assays were performed using 25.0 ng·µL^−1^ (working concentration) DNA and water as control, with 3 replicates. After the reaction, the values of threshold cycles are achieved. Standard curve was established by plotting the threshold cycle (Ct) on the Y-axis and the natural log of concentration (copies·µL^−1^) on the X-axis, and the equation y = k × x + b, coefficient of determination (R^2^) and percentage of variance in copy numbers were achieved.

The total copy number (X_t_) of the *cry1Ac* gene was calculated by relating the Ct value (Y_t_) to the standard curve, then the single cell copy number of the *cry1Ac* gene in the sugarcane samples could be calculated by the following formula:

Copies/genome = X_t_ /[25 × 10^−9^ g × 6.02 × 10^23^/ (genome size of single cell (M bp) × 10^6^ × 660)].

Notes: 25 × 10^−9^ g is the amount of DNA template (measured by UV absorption) used in each reaction system, and 6.02 × 10^23^ is Avogadro's constant. The genome size of sugarcane can be determined by flow cytometry^44^ and the average molecular weight (MW) of a DNA base pair is 660 daltons.

The data are presented as mean values with SE, and one-way repeated measures analysis of variance (ANOVA) was used to test the difference of the single cell copy number of the *cry1Ac* gene in the sugarcane samples. All analyses were conducted with EXCEL 2010 and SPSS 11.5.

### ELISA quantitative detection

Quantification of the Cry1Ac protein of the putative *cry1Ac* sugarcane clones was conducted with quantitative ELISA kits (QuantiPlate™ Kit for Cry1Ab/Cry1Ac, Envirologix, Inc., USA). Cry1Ac standards at concentrations of 0.15625, 0.3125, 0.625, 1.25 and 2.5 ppb were used for calibration. Spectrophotometric measurements were taken at 450 nm with a microtiter plate reader (Biotek, gene, USA). All operations were performed according to the protocol. The data are presented as mean values with SE. Cry1Ac concentration data were analyzed using one-way repeated-measures analysis of variance (ANOVA). Percentage data (protein concentration in sugarcane leaf) were transformed using arcsine [square root (x)] prior to analysis. All analyses were conducted with EXCEL 2010 and SPSS 11.5.

## Author Contributions

Conceived and designed the experiments: D.Z., L.X., Y.Q. Performed the experiments: D.Z., J.G., S.G., Q.L., Q.W. Analyzed the data: D.Z., L.X., Y.Q. Wrote the paper: D.Z., L.X., L.W., Y.Q. Revised and approved the final version of the paper: L.X., Y.Q.

## Supplementary Material

Supplementary Information Supplementary Information in Establishment and application of a loop-mediated isothermal amplification (LAMP) system for detection of cry1Ac transgenic sugarcane
