# Supplementary material for: Establishment and application of a loop-mediated isothermal amplification (LAMP) system for detection of cry1Ac transgenic sugarcane
Source: Sci Rep. 2014 May 9;4:4912. doi: 10.1038/srep04912 (PMC4014978; doi:10.1038/srep04912)
Supplement: Supplementary Information — in Establishment and application of a loop-mediated isothermal amplification (LAMP) system for detection of cry1Ac transgenic sugarcane [file srep04912-s1.pdf]

Establishment and application of a loop-mediated isothermal amplification (LAMP) system for detection of *cry1Ac* transgenic sugarcane

Dinggang Zhou[1], Jinlong Guo[1], Liping Xu[1*], Shiwu Gao[1], Qingliang Lin[1], Qibin Wu[1], Luguang Wu[2], Youxiong Que[1*]

[1] Key Laboratory of Sugarcane Biology and Genetic Breeding, Fujian Agriculture and Forestry University, Ministry of Agriculture, Fuzhou 350002, China

[2] School of Agriculture and Food Sciences, The University of Queensland, Brisbane 4072, Australia.

*Corresponding authors. E-mail: xlpmail@126.com, queyouxiong@hotmail.com

Figure legends

Fig S1. Agarose gel electrophoresis of the plasmid 1Ac0229 PCR products. Lane M: 100 bp DNA ladder. Lanes 1 and 4: ddH_2O (blank control). Lanes 2 and 5: PG0229 PCR products (negative control). Lanes 3 and 6: the plasmid 1Ac0229 PCR products. Lanes 1-3: primers *cry1Ac*-3F and *cry1Ac*-3R. Lanes 4-6: primers F3 and B3.

Fig S2. Effects of 0.80 M betaine on the LAMP reaction. Tubes 1-4: betaine free. Tubes 5-8: 0.80 M betaine. Tubes 1 and 5: ddH_2O. Tubes 2 and 6: FN95-1702 (negative control). Tubes 3, 4, 7 and 8: the plasmid 1Ac0229.

Fig S3. The amplification curves and standard curves obtained in quantitative TaqMan real-time PCR based on the primer pair of *cry1Ac*. In the performed quantitative TaqMan real-time PCR assays, the standard curve formula is y=-3.140 x + 43.622, coefficient of determination (R^2=0.998) and amplification efficiency (E= 1.082).

Fig S4. LAMP primers and their positions in *cry1Ac* gene sequence.

Figures

Figure S1

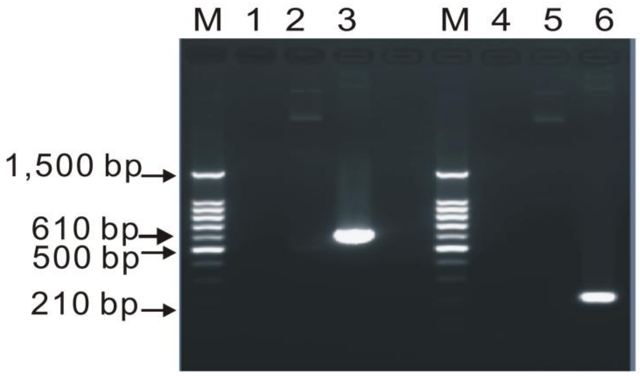

Figure S2

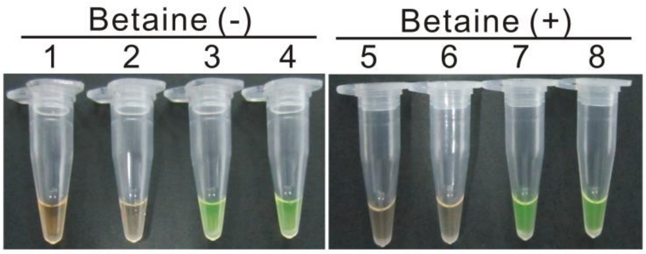

Figure S3

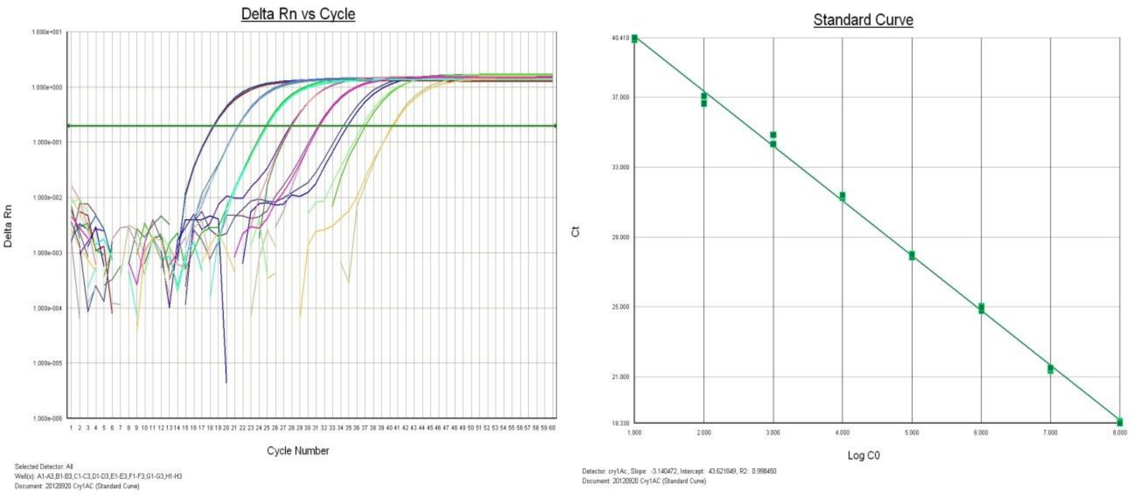
Delta Rn vs Cycle
Delta Rn
Cycle Number
Selected Detector: All
Well(s): A1-A3,B1-B3,C1-C3,D1-D3,E1-E3,F1-F3,G1-G3,H1-H3
Document: 20120920 Cry1AC (Standard Curve)
Standard Curve
Ct
Log C0
Detector: cry1Ac, Slope: -3.140472, Intercept: 43.621849, R2: 0.998450
Document: 20120920 Cry1AC (Standard Curve)

Figure S4

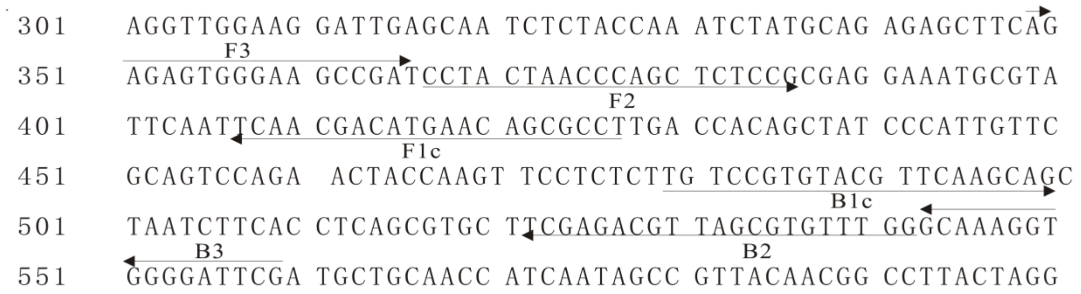
301 AGGTTGGAAG GATTGAGCAA TCTCTACCAA ATCTATGCAG AGAGCTTCAG
F3
351 AGAGTGGGAA GCCGATCCTA CTAACCCAGC TCTCCGCGAG GAAATGCGTA
F2
401 TTCAATTCAA CGACATGAAC AGCGCCTTGA CCACAGCTAT CCCATTGTTC
F1c
451 GCAGTCCAGA ACTACCAAGT TCCTCTCTTG TCCGTGTACG TTCAAGCAGC
B1c
501 TAATCTTCAC CTCAGCGTGC TTCGAGACGT TAGCGTGTTT GGGCAAAGGT
B3
B2
551 GGGGATTCGA TGCTGCAACC ATCAATAGCC GTTACAACGG CCTTACTAGG

Supplementary Tables

Table S1. Estimation of copy number of *cry1Ac* gene in transgenic sugarcane in quantitative TaqMan real-time PCR.

Table S2. Cry1Ac protein content in putative *cry1Ac* transgenic sugarcane by quantitative ELISA detection.

Table S3. The sequence information of LAMP primers used in this experiment.

Tables

Table S1

**Lines**	**Copy number of *cry1Ac* in single cell (Mean ± SD)**
19a-1	21.89 ±0.68 c
19a-3	28.84 ±4.45 cd
19a-5	21.82 ±4.74 c
19b-4	8.98 ±0.55 b
16K-1	75.10 ±1.47 g
16k-3	31.80 ±1.89 d
16k-5	9.32 ±1.44 b
16d-1	110.49 ±1.32 h
16d-2	60.30 ±3.56 f
16d-4	128.14 ±3.20 i
16d-6	46.22 ±2.96 e
20i-2	57.73 ±3.26 f
20i-4	134.77 ±9.12 j
A-2	-
A-5	-
B-2	1.26 ±0.09 a
B-5	-

Notes: "—" means undetected; values in the column followed by the same letters means no significant at P=0.05 level.

Table S2

Lines	Cry1Ac protein/ ng g^{-1} Leaf (Mean ± SD)
FN95-1702	0.00 ±0.09 e
19a-1	445.79 ±0.26 c
19a-3	547.45 ±0.10 a
19a-5	501.78 ±0.44 b
19b-4	42.18 ±0.36 d
ROC22	0.00 ±0.05 o
16K-1	16.87 ±0.02 l
16k-3	24.23 ±0.21 k
16k-5	113.42 ±0.42 f
16d-1	102.56 ±0.08 g
16d-2	87.04 ±0.61 i
16d-4	67.30 ±0.49 j
16d-6	3.86 ±0.17 n
20i-2	90.99 ±0.28 h
20i-4	6.10 ±0.09 m
GT96-44	0.00 ±0.33 p
A-2	0.36 ±0.33 p
A-5	0.18 ±0.33 p
GT94-119	0.00 ±0.33 r
B-2	19.65 ±0.36 q
B-5	0.18 ±0.33 r

Notes: the same letter means no significant at P=0.05 level to its corresponding negative control (FN95-1702, ROC22, GT96-44 and GT94-119).

Table S3

Primer	Position	Primer’s sequences(5'- 3')
F3	349-366	AGAGAGTGGGAAGCCGAT
B3	542-559	CGAATCCCCACCTTTGCC
FIP (F1c+F2)	407-428	AGGCGCTGTTCATGTCGTTGA-
	367-386	CCTACTAACCCAGCTCTCCG
BIP (B1c+B2)	479-499	TGTCCGTGTACGTTCAAGCAG-
	522-542	CCAAACACGCTAACGTCTCGA
